# Etiology and outcomes of fetal renal abnormalities in Southern China: a single-tertiary-center study

**DOI:** 10.1186/s13023-025-03970-3

**Published:** 2025-08-13

**Authors:** Meiying Cai, Yashi Gao, Huili Xue, Xianguo Fu, Hua Cao, Liangpu Xu, Na Lin, Hailong Huang

**Affiliations:** 1 Medical Genetic Diagnosis and Therapy Center, Fujian Maternity and Child Health Hospital College of Clinical Medicine for Obstetrics & Gynecology and Pediatrics, Fujian Medical University, Fujian Key Laboratory for Prenatal Diagnosis and Birth Defect, Fujian Clinical Research Center for Maternal-Fetal Medicine, National Key Obstetric Clinical Specialty Construction Institution of China, Fuzhou, China; 2 Department of Prenatal Diagnosis, Ningde Municipal Hospital, Ningde Normal University, Ningde, China

**Keywords:** Pathogenic variant, Renal abnormality, Prenatal ultrasound, Whole-exome sequencing

## Abstract

**Background:**

Although renal abnormalities are common during fetal growth, the etiology remains largely unclear. This study aimed to determine the outcomes of fetuses with renal anomalies and the corresponding etiologies. We retrospectively analyzed data from 1,019 cases for which chromosomal microarray analysis (CMA) was performed; 58 CMA-negative fetuses were selected for whole-exome sequencing (WES).

**Results:**

Pathogenic copy-number variations were detected in 88 (8.6%) cases, comprising 25 aneuploidies, 10 macrodeletions/macroduplications, and 53 microdeletions/microduplications. Among the latter, abnormalities in the 22q11.2 or 17q12 region were the most common, followed by those in the 16p11.2 region. Of the 58 CMA-negative samples, six showed abnormal WES results. The genes with pathogenic variants were *KMT2D*, *PKD1*, *BBS1*, *NPHP3*, *BBS2*, and *HNF1B*. Hyperechogenic kidney was associated with the highest rate of pathogenic variation (19.8%), followed by renal dysplasia (18.8%). In contrast, hydronephrosis and horseshoe kidney were associated with the lowest incidence of pathogenic variants. The 871 cases with successful follow-up (85.5%) included 120 terminations, 2 stillbirths, and 4 perinatal deaths. Of the remaining 745 live births with renal abnormalities, 63 underwent surgery, and 3 presented with developmental delay. Surgery was most commonly performed in newborns with hydronephrosis (26.8%).

**Conclusions:**

The prenatal ultrasound-screening of fetal renal abnormalities, whether isolated or non-isolated, should be accompanied by rapid etiological analysis. In particular, we noted a high incidence of pathogenic variants in fetal hyperechogenic kidneys, while hydronephrosis was associated with few pathogenic variants and good prognosis after birth.

**Summary:**

The etiology of fetal renal abnormalities remains unclear for many patients. In this study, we investigated the underlying causes, clinical phenotypes, and outcomes. We performed whole-exome sequencing on 1,019 specimens from fetuses with ultrasound-verified renal abnormalities. Our single-tertiary-center study expands on the etiology of renal abnormalities and confirms the clinical utility of whole-exome sequencing for prenatal screening.

## Background

Renal abnormalities are commonly detected during fetal development, with a relatively high incidence compared to other developmental abnormalities [1, 2]. Indeed, renal developmental abnormalities account for more than one-third of prenatal defects [3, 4]. Because the fetal kidney is not essential for maintaining homeostasis, fetuses with renal abnormalities can survive to birth. However, without early postnatal surgical intervention, the abnormality can severely impede renal function and promote the development of comorbidities. Therefore, accurate and rapid prenatal diagnosis of renal abnormalities is important for successful treatment and prognostic evaluation in postnatal management.

Although the cause of most renal abnormalities remains unknown, growing evidence indicates that pathogenic genomic alterations can lead to abnormal kidney development and, thus, a pathologic phenotype [5–8]. Advances in human and mouse genetics have improved our understanding of the pathophysiology of abnormal renal development. For instance, pathogenic variants in genes associated with transcription factors or signaling pathways involved in kidney formation have been associated with developmental defects [9]. However, clinical diagnosis is limited by our incomplete understanding of the genotype−phenotype associations. Further etiological studies are needed to elucidate the pathogenesis of renal abnormalities and improve the accuracy of renal function and prognostic postpartum assessments. In this regard, genetic diagnostics provides a promising tool for individualized treatment, risk-stratified prognosis, and optimized management of renal dysfunction in neonates, and can further improve family counseling and fertility guidance, and minimize unnecessary invasive procedures [10, 11]. In this study, we performed chromosomal microarray analysis (CMA) and whole-exome sequencing (WES) on samples from fetuses with renal abnormalities to determine the possible causes. In addition, pregnancy and follow-up outcomes were evaluated to provide a scientific basis for prenatal counseling and reproductive decision-making.

## Methods

### Study participants

**Fig. 1:**
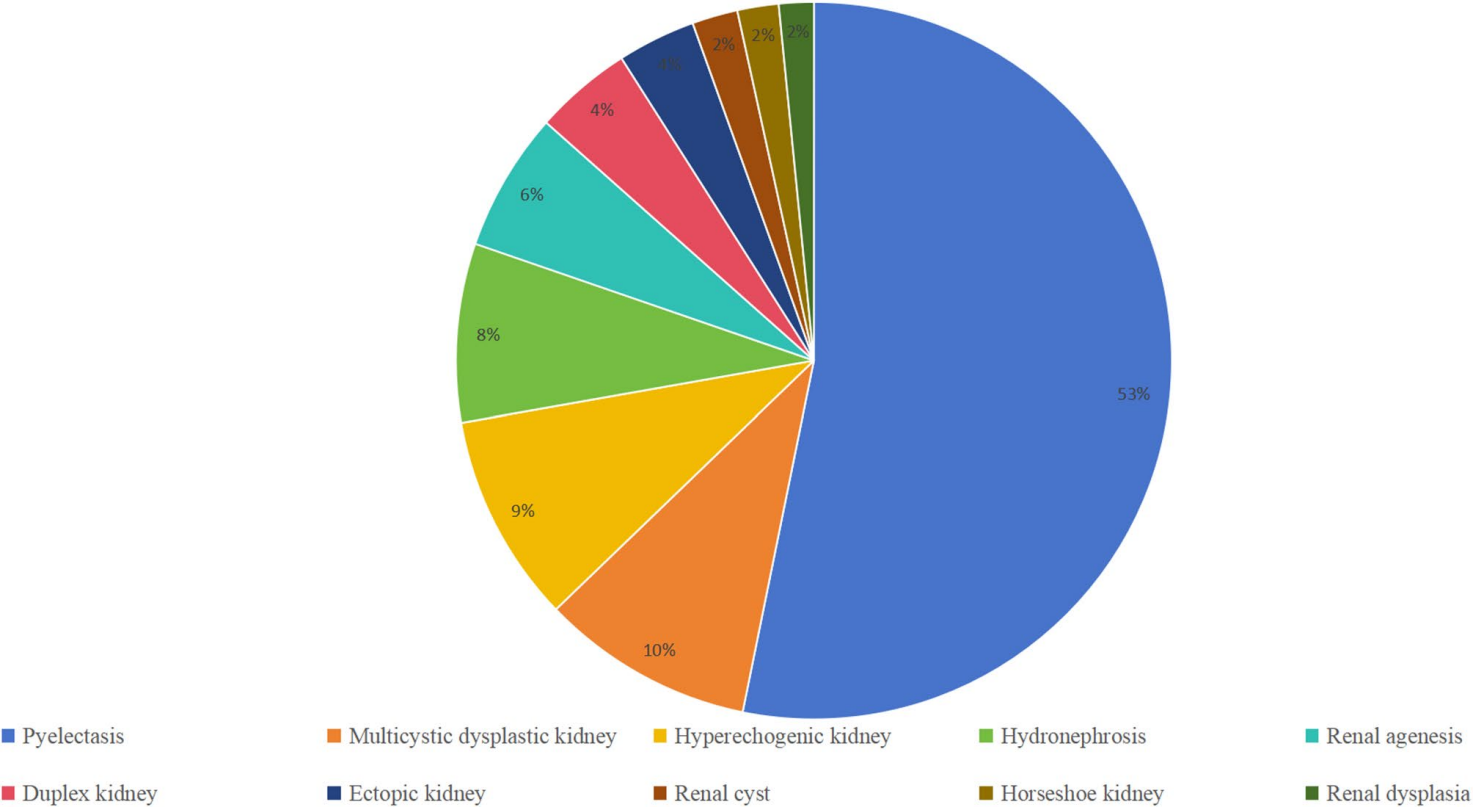
Types of renal anomalies

A retrospective analysis was conducted on data from 1,019 fetuses with renal abnormalities diagnosed via prenatal ultrasound at a single tertiary center in southern China between May 2016 and March 2023. We identified 266 and 753 cases of isolated and non-isolated renal abnormalities, respectively. Among the non-isolated renal abnormalities, 109 cases were complicated with multiple system malformations, 55 with cardiovascular abnormalities, 52 with skeletal system abnormalities, 21 with central nervous system abnormalities, 7 with craniofacial abnormalities, 6 with digestive system abnormalities, and 503 with soft markers on ultrasound (nuchal translucency thickness was the most common, followed by mild tricuspid regurgitation and ventricular echogenic focus). Pyelectasis accounted for more than half of all renal abnormalities (53.2%, 542/1,019), followed by multicystic dysplastic kidney (9.6%, 98/1,019), hyperechogenic kidney (9.4%, 96/1,019), hydronephrosis (8.0%, 82/1,019), renal agenesis (6.3%, 64/1,019, 3 cases with bilateral renal agenesis and 61 with single renal agenesis), duplex kidney (4.4%, 45/1,019), ectopic kidney (3.5%, 36/1,019), renal cyst (2.1%, 21/1,019), horseshoe kidney (1.9%, 19/1,019), and renal dysplasia (at least 1.6%, 16/1,019) (Fig. 1). Fetal samples were obtained using different invasive methods, depending on the gestational week of diagnosis. We collected amniotic fluid samples for 714 and umbilical cord blood samples for 305 fetuses. The median gestational age of the fetuses was 27 (15−37) weeks, and the median age of the pregnant women was 29 (18−42) years at the time the amniotic sac was punctured. The pregnant women had no history of high fever, pregnancy infection, teratogenic substance exposure, or pregnancy-induced hypertension or diabetes. The normal amniotic fluid index (AFI) ranged from 5 to 25 cm, with an AFI greater than 24 cm considered polyhydramnios and less than 5 cm considered oligohydramnios. Anhydramnios refers to an extreme condition during pregnancy where amniotic fluid volume is extremely reduced or even completely absent, and its presence cannot be detected by ultrasound. We identified isolated and non-isolated renal abnormalities—those co-existing with extrarenal abnormalities. This study was approved by the Ethics Committee of the Fujian Maternity and Child Health Hospital (approval No. 2014042). All studies were performed in accordance with relevant guidelines and regulations. Written informed consent was obtained from all parents.

### Prenatal ultrasonography

GE Voluson E8, Philips iU22, Siemens Acuson Sequoia 512, and S2000 Doppler ultrasound systems (transducer frequency range: 2–5 MHz) were used to examine the fetuses, while the mother was in the supine or left lateral decubitus position. A standard fetal anatomical survey was performed of the anatomical planes of the brain, spine, face, abdomen, limbs, placenta, and amniotic fluid, along with a systematic cephalocaudal evaluation. If abnormal kidney development was suspected, the axial, sagittal, and coronal planes of the fetal tissue were observed, and the size, position, renal parenchymal integrity, ureteral dilatation, and bladder morphology and filling status were recorded for both kidneys.

### CMA

Amniotic fluid (10 mL) or cord blood (0.2 mL) was collected, and the genomic DNA was extracted according to the manufacturer’s instructions using a genomic DNA extraction kit (Qiagen, Hilden, Germany). The genomic DNA was analyzed using a CytoScan CMA kit (Affymetrix). Genomic DNA digestion, ligation, amplification, purification, fragmentation, labeling, chip hybridization, washing, scanning, and data analysis were performed following the manufacturer’s protocol (Affymetrix). Fragments with copy-number variations (CNVs) of ≥ 100 kb recognized on a CytoScan™ HD chip with ≥ 90% confidence were selected for analysis. The results were compared with publicly available CNV data, including the Database of Genomic Variants, Children’s Hospital of Philadelphia data repository, Database of Chromosomal Imbalance and Phenotype in Humans using Ensembl Resources, Online Mendelian Inheritance in Man, and University of California Santa Cruz database.

### WES

Optimally sized genome libraries were constructed from the extracted fetal genomic DNA by shearing for exome capture, and exon sequences were obtained using targeted hybridization probes. Bioinformatics analysis of the WES data was performed to screen the potential homozygous and complex heterozygous pathogenic variants as follows: (1) filtering and screening the Human Gene pathogenic variant Database and National Center for Biotechnology Information single nucleotide polymorphism array and excluding uncommon variants exceeding population frequency thresholds (> 0.01) using the Exome Variant Server; (2) excluding deep intronic variants based on predictions from the Berkeley Drosophila Genome Project; (3) excluding silent variants that do not result in amino acid change; and (4) excluding variants whose phenotype is inconsistent with the clinical phenotype of the proband. According to the American College of Medical Genetics and Genomics, pathogenic variants detected using WES are classified into pathogenic, likely pathogenic, variants of uncertain significance, likely benign, and benign. The detected pathogenic variants were verified by Sanger sequencing using DNA from the peripheral blood of the parents (collected on-site).

### Pregnancy outcomes and neonatal follow-up

Follow-ups were performed for all women and fetuses to determine pregnancy outcomes and record postnatal condition, respectively. Follow-up assessments included the recording of postnatal anthropometric parameters (weight/length/head circumference), neurobehavioral development, renal ultrasonography, urinalysis with microscopy, renal function, and surgery consultation.

### Statistical analysis

Data were analyzed using SPSS 25.0 software (IBM SPSS, Armonk, NY, USA), and chi-square tests were used to compare groups. Statistical significance was set at α = 0.05, with *P* < 0.05 indicating significant differences.

## Results

### CMA and WES of fetuses with renal abnormalities

**Fig. 2:**
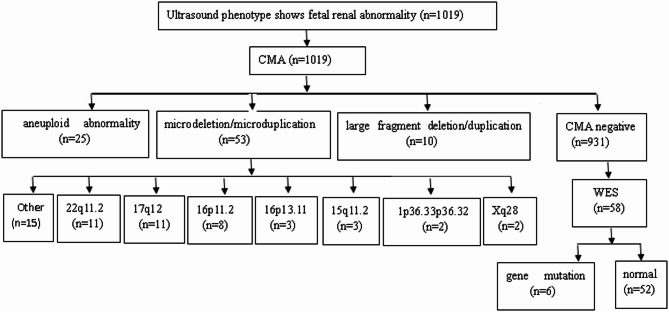
Prenatal genetic diagnosis of renal abnormalities

All 1,019 fetuses with renal abnormalities underwent CMA, and 58 CMA-negative samples were selected for WES (Fig. 2). Of the 1,019 cases, 88 (8.6%) were detected with pathogenic CNVs, which comprised 25 cases with aneuploidies, 10 with macrodeletions/macroduplications, and 53 with microdeletions/microduplications (Fig. 2). Among the 25 cases with aneuploidies, Down syndrome (trisomy 21) was the most common and identified in nine cases, followed by eight cases with 47,XXY (Klinefelter syndrome), six with abnormal mosaic number, and one case each of trisomy 18 syndrome and 45,X (Turner syndrome). Among the 53 cases with microdeletions/microduplications, the most commonly observed abnormalities were related to the 22q11.2 (11 cases; six microdeletions and five microduplications) and 17q12 regions (11 cases), followed by the 16p11.2 (eight cases; seven microdeletions and one microduplication) and 16p13.11 regions (three cases; two microdeletions and one microduplication). Three cases had anomalies in the 15q11.2 region (two cases of microdeletions and one of microduplication) (Table 1).

**Table 1:** Clinical features of fetuses with renal abnormalities and microdeletions or microduplications

Case	CMA	Intrauterine ultrasound phenotype	Including gene	Pathogenic Classification	Size (Mb)	Outcome
1	arr[hg19]22q11.21(18,636,749 − 21,800,471)x1	Pyelectasis, Aberrant right subclavian artery	*USP18*,*DGCR6*,*PRODH*,* DGCR2*,* DGCR14*,* TSSK2*	22q11.21 microdeletion	3.16	TP
2	arr[hg19]22q11.21(18,648,855 − 21,800,471)x1	Bilateral renal agenesis, Tetralogy of fallot, Anhydramnios, Single umbilical artery	*DGCR2*,* TBX1*,* DGCR8*,* DGCR6L*	22q11.21 microdeletion	3.15	TP
3	arr[hg19]22q11.21(18,916,842 − 21,800,471)x1	Pyelectasis, Aberrant right subclavian artery	*DGCR2*,* TBX1*,* DGCR8*,* DGCR6L*	22q11.21 microdeletion	2.9	6 years old, boy, normal
4	arr[hg19]22q11.21(18,916,842 − 21,800,471)x1	Ectopic kidney	*DGCR6*,*DGCR2*,*DGCR14*,*TBX1*,* DGCR8*,* DGCR6L*	22q11.21 microdeletion	2.8	TP
5	arr[hg19]22q11.21(18,631,364 − 21,800,471)x1	Pyelectasis, Ventricular septal defect, Overriding aorta, Pulmonary stenosis	*DGCR6*,*DGCR2*,*DGCR14*,*TBX1*,* DGCR8*,* DGCR6L*	22q11.21 microdeletion	3.1	TP
6	arr[hg19]22q11.21(20,730,143 − 21,800,471)x1	Renal cyst, Choroid plexus cyst, Bilateral talipes equinovarus	*ZNF74*,*SCARF2*,*MED15*,* PI4KA*,* SERPIND1*,*SNAP29*,* CRKL*	22q11.21 microdeletion	1.0	TP
7	arr[hg19]22q11.21(20,730,143 − 21,800,471)x3	Multicystic dysplastic kidney	*ZNF74*,*SCARF2*,*MED15*,* PI4KA*,* SERPIND1*,*SNAP29*,* CRKL*	22q11.21 microduplication	1.0	6 years old, girl, normal
8	arr[hg19]22q11.21(20,730,143 − 21,800,471)x3	Multicystic dysplastic kidney	*ZNF74*,*SCARF2*,*MED15*,* PI4KA*,* SERPIND1*,*SNAP29*,* CRKL*	22q11.21 microduplication	1.0	6 years old, boy, normal
9	arr[hg19]22q11.21(18,636,749 − 21,464,764)x3	Duplex kidney, Parietal bone flattening	*CDC45*,*CLDN5*,*SEPT5*,*GP1BB*,* TBX1*	22q11.21 microduplication	2.8	3 years old, girl, developmental delay
10	arr[hg19]22q11.1q11.21(16,888,900 − 18,644,241)x4	Pyelectasis, Small for gestational age	-	22q11.1q11.21microduplication	1.75	1 years old, girl, normal
11	arr[hg19]22q11.21(20716877_21464764)x3	Pyelectasis, Aberrant right subclavian artery	*CRKL*	22q11.21 microduplication	0.73	Loss follow-up
12	arr[hg19]17q12(34,460,443 − 36,300,630)x1	Hyperechogenic kidney, Left ventriculomegaly	*HNF1B*	17q12 microdeletion	1.48	1 years old, girl, normal
13	arr[hg19]17q12(34,822,465 − 36,243,365)x1	Hyperechogenic kidney	*ZNHIT3*,*MYO19*,*PIGW*,* AATF*,* ACACA*,* TADA2A*,* DUSP14*,*SYNRG*,* DDX52*,*HNF1B*	17q12 microdeletion	1.4	5 years old, boy, normal
14	arr[hg19]17q12(34,822,465 − 36,307,773)x1	Hyperechogenic kidney	*ZNHIT3*,*MYO19*,*PIGW*,* AATF*,* ACACA*,* TADA2A*,* DUSP14*,*SYNRG*,* DDX52*,* HNF1B*	17q12 microdeletion	1.5	5 years old, girl, normal
15	arr[hg19]17q12(34,822,465 − 36,307,773)x1	Hyperechogenic kidney, Choroid plexus cysts, Left ventricular echogenic focus	*HNF1B*	17q12 microdeletion	1.4	5 years old, boy, normal
16	arr[hg19]17q12(34,822,465 − 36,311,009)x1	Multicystic dysplastic kidney, Mild tricuspid regurgitation	*HNF1B*	17q12 microdeletion	1.4	TP
17	arr[hg19]17q12(34,823,294 − 36,410,720)x1	Hyperechogenic kidney, Echogenic bowel	*HNF1B*	17q12 microdeletion	1.58	4 years old, girl, developmental delay
18	arr[hg19]17q12(34822465_36418529)x1	Hyperechogenic kidney, Abnormal ductus venosus flow	*HNF1B*	17q12 microdeletion	1.6	TP
19	arr[hg19]17q12(34822466_36307773)x1	Multicystic dysplastic kidney	*HNF1B*	17q12 microdeletion	1.5	Loss follow-up
20	arr[hg19]17q12(34822492_36307773)x1	Hyperechogenic kidney	*HNF1B*	17q12 microdeletion	1.4	TP
21	arr[hg19]17q12(34822466_36378678)x1	Hyperechogenic kidney	*HNF1B*	17q12 microdeletion	1.4	1 year old, boy, normal
22	arr[hg19]17q12(34822466_36404104)x1	Hyperechogenic kidney, Bilateral talipes equinovarus	*HNF1B*	17q12 microdeletion	1.4	Loss follow-up
23	arr[hg19]16p11.2(28708187_29088624)x1	Pyelectasis, Mild tricuspid regurgitation	*SH2B1*	16p11.2 microdeletion	0.3	2 years old, boy, normal
24	arr[hg19]16p11.2(29428531_30190029)x1	Pyelectasis, Left ventriculomegaly	*TBX6*	16p11.2 microdeletion	0.7	TP
25	arr[hg19]16p11.2(29567297_30350748)x1	Left renal agenesis, Aberrant right subclavian artery	*TBX6*	16p11.2 microdeletion	0.8	TP
26	arr[hg19]16p11.2(28,708,186 − 29,088,624)x1	Renal cyst	*EIF3C*,* ATXN2L*,* TUFM*,* SH2B1*,*ATP2A1*,*RABEP2*,*CD19*,*NFATC2IP*,* SPNS1*,* LAT*	16p11.2 microdeletion	0.4	Miscarriage
27	arr[hg19]16p11.2(29,580,020–30,190,029)x1	Left renal agenesis, Mild tricuspid regurgitation, Echogenic bowel	*TBX6*	16p11.2 microdeletion	0.6	1 year old, boy, normal
28	arr[hg19]16p11.2(29,591,326 − 30,176,508)x1	Bilateral renal agenesis, Bilateral talipes equinovarus	*TBX6*	16p11.2 microdeletion	0.6	TP
29	arr[hg19]16p11.2(29,428,531 − 30,177,916)x1	Hyperechogenic kidney, Single umbilical artery	*BP4-BP5*	16p11.2 microdeletion	0.7	1 year old, boy, normal
30	arr[hg19]16p11.2(28786704_29032280)x3	Right renal agenesis	*ATXN2L*,* TUFM*,* SH2B1*,* ATP2A1*	16p11.2 microduplication	0.2	TP
31	arr[hg19]16p13.12p13.11(14769090_16458424)x1	Duplex kidney, Mild tricuspid regurgitation	*PLA2G10*,* NOMO1*,* MYH11*	16p13.12p13.11microdeletion	1.7	2 years old, girl, normal
32	arr[hg19]16p13.12p13.11(14,780,640 − 16,508,123)x1	Pyelectasis, Left ventricular echogenic focus, Mild tricuspid regurgitation	*NTAN1*,* RRN3*,* KIAA0430*,* NDE1*,* MYH11*	16p13.12p13.11microdeletion	1.5	3 years old, girl, normal
33	arr[hg19]16p13.11(14,892,975 − 16,538,596)x3	Duplex kidney	*NOMO1*,* NPIPA1*,* PDXDC1*,* NTAN1*,* RRN3*	16p13.11 microduplication	1.6	5 years old, girl, normal
34	arr[hg19]15q11.2(22770422_23288350)x1	Pyelectasis, Abnormal ductus venosus flow, Small for gestational age	*TUBGCP5*,* CYFIP1*,* NIPA2*,* NIPA1*	15q11.2 microdeletion	0.5	TP
35	arr[hg19]15q11.2q13.1(23693931_28526905)x3	Pyelectasis, Cleft palate	*SNRPN*,* UBE3A*	15q11.2q13.1 microduplication	4.8	TP
36	arr[hg19]15q11.2(22770422_23625785)x1	Horseshoe kidney	*NIPA1*	15q11.2 microdeletion	0.8	1 year old, boy, normal
37	arr[hg19]Xq28(147550751_155233098)x1	Hyperechogenic kidney, Strong ventricular echo	*F8*,*MECP2*,*RAB39B*	Xq28 microdeletion	7.68	Loss follow-up
38	arr[hg19]Xq28(154,120,632 − 154,564,050)x1	Pyelectasis	*F8*,* MTCP1*,* RAB39B*	Xq28 microdeletion	0.4	4 years old, girl, normal
39	arr[hg19]1p36.33p36.32(849467_4894800)x1	Hyperechogenic kidney, Ventriculomegaly, Mild tricuspid regurgitation	*GABRD*,* GNB1*	1p36.33p36.32 microdeletion	4.0	Loss follow-up
40	arr[hg19]1p36.33p36.32(849,466-4,894,800)x1	Hyperechogenic kidney, Ventriculomegaly	*GABRD*,* GNB1*	1p36.33p36.32 microdeletion	4.0	TP
41	arr[hg19]1q21.1q21.2(146023923–147830830)x1	Pyelectasis, Small for gestational age, Mild tricuspid regurgitation	*GJA5*,* GJA8*	1q21.1q21.2 microdeletion	1.8	2 years old, boy, developmental delay
41	arr[GRCh37]18q21.2(53140502–53421172)x1	Pyelectasis, Ventricular echogenic focus	*TCF4*	18q21.2 microdeletion	0.27	3 years old, girl, normal
42	arr[hg19]22q11.23(23654064–25041592)x3	Pyelectasis, Choroid plexus cysts, Mild tricuspid regurgitation	*SMARCB1*	22q11.23 microduplication	1.4	1 year old, boy, normal
43	arr[hg19]6p21.32p21.31(32,965,747 − 35,234,269)x3	Pyelectasis, Ventricular echogenic focus	*COL11A2和SYNGAP1*	6p21.32p21.31microduplication	2.2	5 years old, boy, normal
44	arr[hg19]4q31.3q32.2(155,463,038–162,158,990)x1	Ectopic kidney, Echogenic bowel	*LRAT*,* GRIA2*,* GLRB*,* ETFDH*	4q31.3q32.2 microdeletion	6.7	TP
45	arr[hg19]22q13.31q13.33(46444129–51197766)x1	Multicystic dysplastic kidney, Small for gestational age	*SHANK3*	22q13.31q13.33 microdeletion	4.7	TP
46	arr[hg19]17p12(14,083,054 − 15,482,833)x1	Left renal agenesis	-	17p12 microdeletion	1.4	Loss follow-up
47	arr[hg19]7q11.23(72,701,098 − 74,069,645)x3	Left renal agenesis, Ventricular septal defect	-	7q11.23 microduplication	1.3	TP
48	arr[hg19]16q23.2q24.3(79,800,878 − 90,146,366) hmz,	Left renal agenesis, Ventricular septal defect, Aortic stenosis, Echogenic bowel	UPD	16q23.2q24.3loss of heterozygosity	10.3	TP
49	arr[hg19]15q13.2q13.3(30913573–32444261)x1	Duplex kidney, Ventriculomegaly	*ARHGAP11B*,* FAN1*,* TRPM1*,* MIR211*,* KLF13*,* OTUD7A*,* CHRNA7*	15q13.2q13.3 microdeletion	1.5	TP
50	arr[hg19]17q24.3q25.1(70,731,144 − 72,248,511)x4	Ectopic kidney, Hygroma colli, Single umbilical artery	-	17q24.3q25.1 microduplication	1.5	TP
51	arr[hg19]16p12.2(21816543–22441367)x1	Renal dysplasia, Ventriculomegaly, Ventricular echogenic focus	*OTOA*,* UQCRC2*,* EEF2K*,* CDR2*	16p12.2 microdeletion	0.6	2 years old, girl, normal
52	arr[hg19]Xp22.31(6455151–8135568)x0	Pyelectasis, Small for gestational age, Ventricular echogenic focus, Mild tricuspid regurgitation	*PUDP*,* STS*,* VCX*,* PNPLA4*	Xp22.31 microdeletion	1.7	TP
53	arr[hg19]4p16.3(68345-3216703)x1,	Renal dysplasia, Small for gestational age, Ventricular septal defect, Nasal hypoplasia, Abnormal ductus venosus flow, Mild tricuspid regurgitation	*ZNF141*,* PIGG*,* PDE6B*,* ATP5I*,* MYL5*	4p16.3 microdeletion	3.2	TP

TP, termination of pregnancy; UPD, uniparental disomy

Six samples among the 58 CMA-negative samples (10.3%) showed abnormal WES results. Pathogenic variants were detected in *KMT2D*, *PKD1*, *BBS1*, *NPHP3*, *BBS2*, and *HNF1B* (Table 2).

**Table 2:** Clinical characteristics of single-gene pathogenic variants in fetuses with renal abnormalities

Case	Intrauterine ultrasound phenotype	WES	Gene	Mode of inheritance	Origin	ACMG variation classification	Outcome
1	Hyperechogenic kidney, Ventriculomegaly, Oligoamnios	Chr12:49434993 NM_003482.4 c.6547_6560del(p.Y2183Pfs*14)	*KMT2D*	AD	denovo	P (PVS1, PS2, PM2_Supporting)	TP
2	Multicystic dysplastic kidney, Small for gestational age	Chr16:2140196 NM_001009944.3 c.12445-1G > C	*PKD1*	AD	unknown	LP (PVS1_Strong, PS1_Supporting, PM2_Supporting)	Miscarriage
3	Multicystic dysplastic kidney	Chr16:2140337–2,140,339 NM_001009944 c.12391_12393del(p.E4131del)	*PKD1*	AD	unknown	LP (PM1, PM4, PP4, PP1_Moderate, PM2_Supporting)	2 years old, girl, normal
4	Multicystic dysplastic kidney, Oligoamnios	chr11:66293660 NM_024649.4 c.1177 C > T(p.Arg393*)	*BBS1*	AR	Parent	P (PVS1 + PM2 + PM3_Supporting)	TP
5	Hyperechogenic kidney, Oligoamnios, Ventriculomegaly	Chr3:132403565,Chr3:132438657 NM_153240 c.3402_3403del(p.A1135Sfs*5),c.411delT(p.Q138Rfs*11) Chr16:56530975,Chr16:56544770 NM_031885 c.1814 C > G(p.S605*),c.534 + 1G > T	*NPHP3* *BBS2*	AR AR	Parent Parent	P (PVS1, PM3, PM2_Supporting) P (PVS1, PM3_Strong, PM2_Supporting)	TP
6	Hyperechogenic kidney	Chr17:36064918–36,064,921 NM_000458 c.1339 + 3_1339 + 6delAAGT	*HNF1B*	AD	denovo	LP (PS2,PM2_Supporting)	3 months old, girl, normal

ACMG, The American College of Medical Genetics and Genomics; P, pathogenic; LP, likely pathogenic; AD, autosomal dominant; AR: autosomal recessive; TP, termination of pregnancy

### Frequency of pathogenic variation among groups

Pathogenic CNVs were detected in 88 (8.6%) fetuses. Pathogenic genes were detected in 6 (10.3%, 6/58) of the 58 CMA-negative fetuses. A similar amount of pathogenic CNVs were detected in isolated (9.0%, 24/266) and non-isolated cases (8.5%, 64/753; *P* = 0.80). A similar amount of pathogenic genes were detected in isolated (10.7%, 3/28) and non-isolated cases (10.0%, 3/30; *P* = 1.00). A similar amount of pathogenic CNVs were detected in unilateral (10.5%, 28/266) and bilateral cases (8.0%, 60/753; *P* = 0.21). In contrast, pathogenic variants were detected exclusively in bilateral cases (17.6%, 6/34; *P* = 0.032 vs. unilateral cases).

Pathogenic variants were detected at different frequencies among the abnormal renal phenotypes (Table 3) but were mostly associated with oligohydramnios (50.0%, 3/6), followed by hyperechogenic kidney (19.8%, 19/96), renal dysplasia (18.8%, 3/16), and renal agenesis (17.2%, 11/64). Pathogenic variations were detected at much lower rates in fetuses with hydronephrosis and horseshoe kidney [3.7% (3/82) and 5.3% (1/19), respectively].

**Table 3:** Pathogenic genome detection of fetuses with different types of renal abnormalities

Classified	Number of cases	Number of pathogenic genome cases	Detection rate(%)
Pyelectasis	542	33	6.1
Isolated Pyelectasis	61	3	4.9
Non-isolated Pyelectasis	481	30	6.2
Unilaterality Pyelectasis	21	2	9.5
Bilaterality Pyelectasis	521	31	6.0
Multicystic dysplastic kidney	98	14	14.3
Isolated Multicystic dysplastic kidney	56	6	10.7
Non-isolated Multicystic dysplastic kidney	42	8	19.0
Unilaterality Multicystic dysplastic kidney	84	5	6.0
Bilaterality Multicystic dysplastic kidney	14	9	64.3
Hyperechogenic kidney	96	19	19.8
Isolated Hyperechogenic kidney	14	2	14.3
Non-isolated Hyperechogenic kidney	82	17	20.7
Unilaterality Hyperechogenic kidney	8	1	12.5
Bilaterality Hyperechogenic kidney	88	18	20.5
Hydronephrosis	82	3	3.7
Isolated Hydronephrosis	42	1	2.4
Non-isolated Hydronephrosis	40	2	5.0
Unilaterality Hydronephrosis	66	2	3.0
Bilaterality Hydronephrosis	16	1	6.3
Renal agenesis	64	11	17.2
Isolated Renal agenesis	33	5	15.2
Non-isolated Renal agenesis	31	6	19.4
Unilaterality Renal agenesis	62	9	14.5
Bilaterality Renal agenesis	2	2	100
Duplex kidney	45	5	11.1
Isolated Duplex kidney	19	2	10.5
Non-isolated Duplex kidney	26	3	11.5
Unilaterality Duplex kidney	44	5	11.4
Bilaterality Duplex kidney	1	0	0
Ectopic kidney	36	3	8.3
Isolated Ectopic kidney	20	2	10.0
Non-isolated Ectopic kidney	16	1	6.3
Unilaterality Ectopic kidney	34	2	5.9
Bilaterality Ectopic kidney	2	1	50
Renal cyst	21	2	9.5
Isolated Renal cyst	10	1	10.0
Non-isolated Renal cyst	11	1	9.1
Unilaterality Renal cyst	21	2	9.5
Bilaterality Renal cyst	0	0	0
Horseshoe kidney	19	1	5.3
Isolated Horseshoe kidney	7	0	0
Non-isolated Horseshoe kidney	12	1	8.3
Unilaterality Horseshoe kidney	0	0	0
Bilaterality Horseshoe kidney	19	1	5.3
Renal dysplasia	16	3	18.8
Isolated Renal dysplasia	4	0	0
Non-isolated Renal dysplasia	12	3	21.4
Unilaterality Renal dysplasia	9	0	0
Bilaterality Renal dysplasia	7	3	42.9
Oligohydramnios	6	3	50

### Pregnancy outcomes and postpartum follow-up of fetuses with renal abnormalities

Of the 1,019 fetuses, follow-up information was available for 871 (85.5%), including 120 terminated pregnancies (25 with aneuploidies, 10 with large fragment deletions/duplications, 23 with microdeletions/microduplications, and 4 with pathogenic variants), 2 stillbirths, and 4 perinatal deaths (Table 4). Among the 745 live births with follow-up information, surgery due to clinical symptoms was performed in 63 cases, and developmental delay was recorded in three cases with pathogenic CNVs. The remaining 679 newborns did not present with abnormal phenotypes until the date of data collection. Among all live births, surgery was most often performed for hydronephrosis (26.8%, 22/82), followed by ectopic kidney (22.2%, 8/36) and duplex kidney (20.0%, 9/45; Table 4). Normal phenotypes were typically observed among live births with pyelectasis (78.0%, 423/542), followed by those with horseshoe kidney (73.7%, 14/19) and duplex kidney (66.7%, 30/45).

**Table 4:** Follow-up of fetuses with different types of renal abnormalities

Classified	Loss follow-up	TP	Stillbirth	Follow-up Postnatal death	Surgery	Developmental retardation	Normal phenotype
Pyelectasis	70 (12.9%)	37 (6.8%)	0 (0%)	2 (0.4%)	10 (1.8%)	1 (0.2%)	422 (77.9%)
Isolated Pyelectasis	8 (13.1%)	1 (1.6%)	0 (0%)	0 (0%)	0 (0%)	0 (0%)	52 (85.2%)
Non-isolated Pyelectasis	62 (12.9%)	36 (7.5%)	0 (0%)	2 (0.4%)	10 (2.1%)	1 (0.2%)	370 (76.9%)
Unilaterality Pyelectasis	7 (33.3%)	4 (19.0%)	0 (0%)	0 (0%)	5 (23.8%)	0 (0%)	5 (23.8%)
Bilaterality Pyelectasis	63 (12.1%)	33 (6.3%)	0 (0%)	2 (0.4%)	5 (1.0%)	1 (0.2%)	417 (80.0%)
Multicystic dysplastic kidney	18 (18.4%)	21 (21.4%)	0 (0%)	0 (0%)	11 (11.2%)	0 (0%)	48 (49.0%)
Isolated Multicystic dysplastic kidney	13 (23.2%)	8 (14.3%)	0 (0%)	0 (0%)	4 (7.1%)	0 (0%)	31 (55.4%)
Non-isolated Multicystic dysplastic kidney	5 (11.9%)	13 (31.0%)	0 (0%)	0 (0%)	7 (16.7%)	0 (0%)	17 (40.5%)
Unilaterality Multicystic dysplastic kidney	17 (20.2%)	9 (10.7%)	0 (0%)	0 (0%)	10 (11.9%)	0 (0%)	48 (57.1%)
Bilaterality Multicystic dysplastic kidney	1 (7.1%)	12 (85.7%)	0 (0%)	0 (0%)	1 (7.1%)	0 (0%)	0 (0%)
Hyperechogenic kidney	16 (16.7%)	27 (28.1%)	1 (1.0%)	1 (1.0%)	2 (2.1%)	1 (1.1%)	48 (50.0%)
Isolated Hyperechogenic kidney	3 (21.4%)	0 (0%)	0 (0%)	0 (0%)	1 (7.1%)	0 (0%)	10 (71.4%)
Non-isolated Hyperechogenic kidney	13 (15.9%)	27 (32.9%)	1 (1.2%)	1 (1.2%)	1 (1.2%)	1 (1.2%)	38 (46.3%)
Unilaterality Hyperechogenic kidney	3 (37.5%)	2 (25.0%)	0 (0%)	0 (0%)	1 (12.5%)	0 (0%)	2 (25.0%)
Bilaterality Hyperechogenic kidney	13 (14.8%)	25 (28.4%)	1 (1.1%)	1 (1.1%)	1 (1.1%)	1 (1.1%)	46 (52.3%)
Hydronephrosis	15 (18.3%)	4 (4.9%)	0 (0%)	1 (1.2%)	22 (26.8%)	0 (0%)	40 (48.8%)
Isolated Hydronephrosis	4 (9.5%)	1 (2.4%)	0 (0%)	0 (0%)	13 (31.0)	0 (0%)	24 (57.1%)
Non-isolated Hydronephrosis	11 (27.5%)	3 (7.5%)	0 (0%)	1 (2.5%)	9 (22.5%)	0 (0%)	16 (40.0%)
Unilaterality Hydronephrosis	13 (19.7%)	3 (4.5%)	0 (0%)	1 (1.5%)	18 (27.3%)	0 (0%)	31 (47.0%)
Bilaterality Hydronephrosis	2 (12.5%)	1 (6.3%)	0 (0%)	0 (0%)	4 (25.0%)	0 (0%)	9 (56.3%)
Renal agenesis	7 (10.9%)	18 (28.1%)	0 (0%)	0 (0%)	1 (1.6%)	0 (0%)	38 (59.4%)
Isolated Renal agenesis	6 (18.2%)	3 (9.1%)	0 (0%)	0 (0%)	0 (0%)	0 (0%)	24 (72.7%)
Non-isolated Renal agenesis	1 (3.2%)	15 (48.4%)	0 (0%)	0 (0%)	1 (3.2%)	0 (0%)	14 (45.2%)
Unilaterality Renal agenesis	7 (11.3%)	16 (25.8%)	0 (0%)	0 (0%)	1 (16.1%)	0 (0%)	38 (61.3%)
Bilaterality Renal agenesis	0 (0%)	2 (100%)	0 (0%)	0 (0%)	0 (0%)	0 (0%)	0 (0%)
Duplex kidney	3 (6.7%)	3 (6.7%)	0 (0%)	0 (0%)	9 (20.0%)	1 (2.2%)	29 (64.4%)
Isolated Duplex kidney	0 (0%)	1 (5.3%)	0 (0%)	0 (0%)	5 (26.3%)	0 (0%)	13 (68.4%)
Non-isolated Duplex kidney	3 (11.5%)	2 (7.7%)	0 (0%)	0 (0%)	4 (15.4%)	1 (3.8%)	16 (61.5%)
Unilaterality Duplex kidney	3 (6.8%)	3 (6.8%)	0 (0%)	0 (0%)	9 (20.5%)	1 (22.7%)	28 (63.6%)
Bilaterality Duplex kidney	0 (0%)	0 (0%)	0 (0%)	0 (0%)	0 (0%)	0 (0%)	1 (100%)
Ectopic kidney	6 (16.7%)	3 (8.3%)	0 (0%)	0 (0%)	8 (22.2%)	0 (0%)	19 (52.8%)
Isolated Ectopic kidney	2 (10.0%)	2 (10.0%)	0 (0%)	0 (0%)	4 (20.0%)	0 (0%)	12 (60.0%)
Non-isolated Ectopic kidney	4 (25.0%)	1 (6.3%)	0 (0%)	0 (0%)	4 (25.0%)	0 (0%)	7 (43.8%)
Unilaterality Ectopic kidney	5 (14.7%)	2 (5.9%)	0 (0%)	0 (0%)	8 (23.5%)	0 (0%)	19 (55.9%)
Bilaterality Ectopic kidney	1 (50.0%)	1 (50.0%)	0 (0%)	0 (0%)	0 (0%)	0 (0%)	0 (0%)
Renal cyst	5 (23.8%)	2 (9.5%)	1 (4.8%)	0 (0%)	0 (0.0%)	0 (0%)	13 (61.9%)
Isolated Renal cyst	1 (10.0%)	1 (10.0%)	1 (10.0%)	0 (0%)	0 (0%)	0 (0%)	7 (70.0%)
Non-isolated Renal cyst	4 (36.4%)	1 (9.1%)	0 (0%)	0 (0%)	0 (0%)	0 (0%)	6 (54.5%)
Unilaterality Renal cyst	5 (23.8%)	2 (9.5%)	1 (4.8%)	0 (0%)	0 (0.0%)	0 (0%)	13 (61.9%)
Bilaterality Renal cyst	0 (0%)	0 (0%)	0 (0%)	0 (0%)	0 (0%)	0 (0%)	0 (0%)
Horseshoe kidney	3 (15.8%)	2 (10.5%)	0 (0%)	0 (0%)	0 (0.0%)	0 (0%)	14 (73.7%)
Isolated Horseshoe kidney	0 (0%)	0 (0%)	0 (0%)	0 (0%)	0 (0%)	0 (0%)	7 (100.0%)
Non-isolated Horseshoe kidney	3 (25.0%)	2 (16.7%)	0 (0%)	0 (0%)	0 (0%)	0 (0%)	7 (58.3%)
Unilaterality Horseshoe kidney	0 (0%)	0 (0%)	0 (0%)	0 (0%)	0 (0%)	0 (0%)	0 (0%)
Bilaterality Horseshoe kidney	3 (15.8%)	2 (10.5%)	0 (0%)	0 (0%)	0 (0.0%)	0 (0%)	14 (73.7%)
Renal dysplasia	5 (31.3%)	3 (18.8%)	0 (0%)	0 (0%)	0 (0.0%)	0 (0%)	8 (50.0%)
Isolated Renal dysplasia	2 (50.0%)	0 (0%)	0 (0%)	0 (0%)	0 (0%)	0 (0%)	2 (50.0%)
Non-isolated Renal dysplasia	3 (25.0%)	3 (25.0%)	0 (0%)	0 (0%)	0 (0%)	0 (0%)	6 (50.0%)
Unilaterality Renal dysplasia	3 (33.3%)	1 (11.1%)	0 (0%)	0 (0%)	0 (0%)	0 (0%)	5 (55.6%)
Bilaterality Renal dysplasia	2 (28.6%)	2 (28.6%)	0 (0%)	0 (0%)	0 (0%)	0 (0%)	3 (42.9%)
Oligohydramnios	0 (0%)	5 (83.3%)	0 (0%)	1 (16.7%)	0 (0%)	0 (0%)	0 (0%)

## Discussion

The kidney plays an important role in normal development and maintenance of physiological function. During fetal development, metabolites are received through the placenta via the mother, thus abnormal kidney development does not affect normal development. Kidney defects only become apparent once the newborn exhibits independent excretory function. The most common renal abnormalities are hydronephrosis, duplex kidney, renal cysts, renal agenesis, and multicystic dysplastic kidney. Because the degree and presence of abnormalities in both kidneys can seriously affect newborn survival, timely prenatal ultrasonography is essential to confirm the disease type and implement targeted interventions. The spectrum of renal abnormalities is broad, ranging from pyelectasis to severe bilateral renal agenesis. This study identified 10 types of fetal renal abnormalities using prenatal ultrasound in 1,019 cases. The top three were pyelectasis followed by multicystic dysplastic kidney, and hyperechogenic kidney. In contrast, the prevalences of horseshoe kidney and renal dysplasia were relatively low.

Accumulating data show that approximately one-third of renal abnormalities are attributed to genetic changes, such as CNVs and new pathogenic variants. Weber et al. [12] tested 30 patients with urinary system malformations and reported pathogenic CNVs in 10% of them, with pathogenic fragments in the 1q21.1, 2q37.1-q37.3, 3q23-q25.1, and 7q36.2-q36.3 regions. Sanna-Cherchi et al. [13] tested a large cohort of patients with abnormal kidney development and detected CNVs in 10.5% of them. Caruana et al. [14] detected pathogenic CNVs in 3.9% of patients with congenital anomalies of the kidneys and urinary tract. Fu et al. [15] tested 30 fetal samples with normal karyotype indicated by prenatal ultrasound as multicystic dysplastic kidney and detected pathogenic CNVs in 16.7% of them. Xi et al. [16] tested 37 fetal samples, also indicated by prenatal ultrasound as multicystic dysplastic kidney with normal karyotype, and identified pathogenic CNVs in 13.5% of them. In this study, pathogenic CNVs were detected in 8.6% (88/1,019) of samples, which is largely consistent with previous reports. Further, we found that CNVs may be a common pathogenic factor associated with renal abnormalities. Microdeletions in 17q12 and 22q11.2 have been identified as the most common CNVs in renal abnormalities [13]. This study also showed that fetal renal dysplasia was most commonly associated with 22q11.2 microdeletions/microduplications and 17q12 microdeletions. The pathogenic *HNF1B* gene in the 17q12 region is linked to renal abnormalities and plays an important role in the embryonic development of the kidney, pancreas, and liver [17–19]. *HNF1B* variations can lead to abnormal kidney development [20]. Nearly 40% of patients with 22q11.2 microdeletions have renal abnormalities [13]. Different gene impairments in the 22q11.2-microdeletion-syndrome region will result in different phenotypes, such as developmental abnormalities, metabolic diseases, and immune deficiencies. In this study, 16p11.2 microdeletions were also relatively common, detected in seven fetuses with renal abnormalities. 16p11.2 microdeletions mainly manifest as a multisystem involvement, with clinical symptoms including autism spectrum disorder, intellectual disability, developmental delay, epilepsy, and spinal deformity; some patients are also at risk of obesity [21, 22]. At present, no pathogenic genes related to renal abnormalities have been identified in patients with 16p11.2 microdeletions. In this study, the intrauterine ultrasound phenotype of fetuses with renal abnormalities carrying 16p11.2 microdeletions was inconsistent with the general manifestations. Whether renal abnormalities in fetuses are related to 16p11.2 microdeletions requires further investigation using a larger number of cases and multicenter datasets.

Defects in single genes can lead to renal abnormalities [23, 24]. Chatterjee et al. [25] tested 122 patients with renal abnormalities and found that 5% of them harbored rare and new pathogenic variants. Saisawat et al. [26] found *TRAP1* pathogenic variants in families with renal abnormalities. Vivante et al. [27] found recessive pathogenic variants in nine of 33 families with renal abnormalities. Humbert et al. [28] detected *ITGA8* pathogenic variants in several families with bilateral kidney agenesis. In this study, WES of samples from 58 fetuses with renal malformations but without abnormal CMA results detected pathogenic variants in six fetuses. All the pathogenic variants in our study (*KMT2D*, *PKD1*, *BBS1*, *NPHP3*, *BBS2*, and *HNF1B*) have been associated with kidney development [29–34]. These results demonstrate the applicability of WES for detecting pathogenic genetic causes in unexplained fetal renal abnormalities, which can ultimately inform prenatal management, prognostic assessment, and pregnancy status, and enable pre-embryo-transfer diagnoses for families with recurrent congenital structural malformations.

Pathogenic variants were detected at similar rates between the group with isolated renal abnormalities and that with co-existing ultrasound abnormalities. Pathogenic genes were more common in bilateral than in unilateral cases. Previous studies suggested that renal defects co-existing with malformations in other systems are likely associated with pathogenic variants [35]. The inconsistency between this and previous studies may be due to a bias in the different study groups. Pathogenic variants were detected at different frequencies among the various types of renal abnormalities but were most commonly associated with oligohydramnios, followed by hyperechogenic kidney, renal dysplasia, renal agenesis, horseshoe kidney, and hydronephrosis. Previous studies also reported that pathogenic genomic abnormalities are most common in hyperechogenic kidney and less common in hydronephrosis [35]. The high frequency of pathogenic variants among cases with hyperechogenic kidneys may reflect the close relationship of this presentation with various fetal diseases, often accompanied by fetal chromosome abnormalities, such as Perlman and Beckwith–Wiedemann syndrome [36, 37]. Therefore, the presence of fetal hyperechogenic kidney at prenatal examination warrants further systematic imaging to address the risk of genomic abnormalities. We detected few pathogenic variants in isolated fetal pelviectasis (4.9%), consistent with a previous report [38]. The majority (64–94%) of fetal pelviectasis cases represent physiological phenomena that typically resolve spontaneously during late gestation or postnatally [39]. These collective findings suggest that isolated pelviectasis may not justify routine CMA testing.

Follow-up was successful in 85.5% of the cases (871/1,019). After counseling, 120 pregnancies were terminated, including 25 cases of aneuploidies, 10 of macrodeletions/macroduplications, 23 of microdeletions/microduplications, and 4 of pathogenic variants. The clinical phenotypes of patients with microdeletions/microduplications are diverse and mainly include multiple deformations, special facial features, mental disabilities, and developmental delay; multisystem abnormalities often exist simultaneously [40, 41]. We found that 21 fetuses with microdeletions/microduplications associated with renal abnormalities had no abnormal phenotypes and did not require postnatal surgery. In addition, one child with a 22q11.21 microduplication, 17q12 microdeletion, and 1q21.1q21.2 microdeletion linked to renal abnormalities presented with developmental delay after birth. These deviations may be related to the young age of the study participants and aberrant development of the nervous system, among other abnormal phenotypes; thus, further follow-up is needed. Bilateral renal agenesis is the most severe condition among fetal kidney abnormalities. Although the incidence is very low, no survivors have been documented. We found only three cases with bilateral renal agenesis, for which pregnancy was terminated. Renal abnormalities can be effectively managed by surgical treatment after birth, with minimal impact on the life of the newborn [42–44]. Of the 745 live births that were examined by specialists, 63 newborns with renal abnormalities received surgical treatment due to clinical symptoms, and were in good condition. Surgical treatment was most commonly performed in hydronephrosis (26.8%, 22/82). Hydronephrosis can resolve on its own in more than 70% of cases [45]. In this study, 60 (75.0%, 60/82) newborns with hydronephrosis returned to normal or improved. The adverse pregnancy rate of hydronephrosis is low, the prognosis is good, and, if necessary, surgical treatment is effective. With the rapid development of prenatal diagnosis, the detection rate of fetal renal abnormalities has also increased, and can be used to validate pregnancy termination criteria. After a prenatal diagnosis of renal abnormalities, the pregnant women and their families should be informed of the prognosis to avoid unnecessary suffering.

This study has some limitations. First, the sample size for WES was small, and the excluded cases may carry pathogenic variants. Second, the follow-up time was short, and some clinical phenotypes could not be determined. In future work, we intend to continue tracking the same individuals to elaborate on the clinical data for more detailed developmental analyses. Further, the decreasing cost of WES technologies can support the establishment of high-resolution databases for long-term multiregional studies and the development of accurate diagnostic/prognostic criteria.

## Conclusions

Prenatal etiological diagnosis should be considered for all fetal renal abnormalities detected by ultrasound, whether isolated or non-isolated, with the exception of isolated pelviectasis. With normal CMA results, WES may be used as a complementary test to prevent certain unmanifested monogenic inherited diseases, especially given the high risk of fetal hyperechogenic kidney. In contrast, hydronephrosis has a much lower risk and can be successfully managed with postnatal surgery. This complementary approach can help avoid the unnecessary termination of pregnancy in vague and complex cases.

## Data Availability

All data on which the findings of this study are based are provided within the manuscript.

## Abbreviations

CMA: Chromosomal microarray analysis
WES: Whole-exome sequencing
AFI: Normal amniotic fluid index
CNV: Copy-number variation
